# Melange

**Published:** 1885-11

**Authors:** 


### MELANGE.

THE SORRY BIRDS OF THE MEDICAL NEST after having befouled it, now propose to leave it for a cleaner one—one "so honorable" that none (others) shall enter thérein—no not one—except he be of the sorry bird persuasion, and shall have contributed his share to the slime with which the old nest has been befouled; and shall have "declined" to hold any office in the Congress as "now proposed" to be organized. "The American medical profession are a great profession ; we are the American medical profession."—*Hays, Shrady & Co.*

A NEW NAME for an old disease, the "Demdengue."

OUR TURN.—Observing our notice of Dr. Cook's marriage in last issue, a subscriber writes us : "Dear Doctor, when it comes *your* turn, don't spell it 'Hymeneal.'" How shall we spell it brother? We don't want to have a *bad spell* of it when it *does* come our "turn."

THE COLUMBUS MAN writes us that "there will not be left a *greasy spot* of the International Congres." He avers, however, that he wasn't thinking of "the fat in the fire" when he wrote it. Oh, my — or rather, Oh our! — was there ever such an in-de-*fat*-igable greaser? No, Doctor, there will *not* be, so long as *lye* will neutralize grease. Let soap from the amount of the *former article* that has been manufactured there will be an *emulsion*.

JOURNAL CHANGES.—From and after January, 1886, the well known *Epitome of American Medicine and Surgery*, published by Townsend, will be changed to a monthly.

